# Supplementary figures and images for: Interrogating the plasma proteome of repetitive head impact exposure and chronic traumatic encephalopathy
Source: Mol Neurodegener. 2025 Jun 16;20:71. doi: 10.1186/s13024-025-00860-x (PMC12168330; doi:10.1186/s13024-025-00860-x)

**A**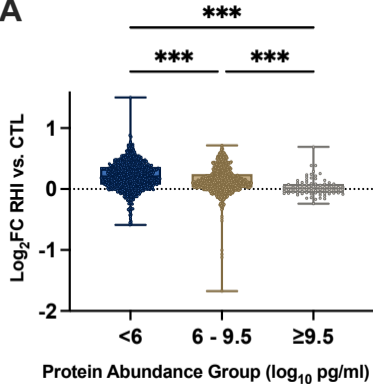**B**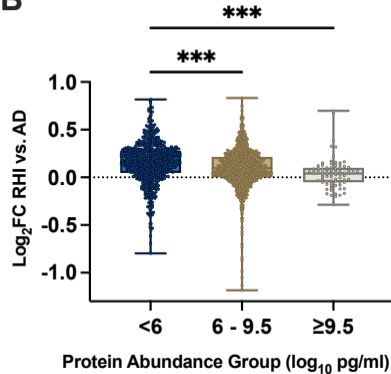**C**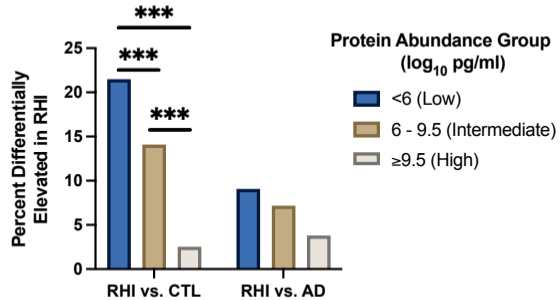

Supplement: Supplementary file 1 — Additional file 1: Supplementary Table 1. Plasma protein differential abundance across RHI, AD, and CTL. Supplementary Table 2. Plasma network module membership. Supplementary Table 3. Plasma network module gene ontologyenrichment. Supplementary Table 4. Plasma network module eigenprotein differences across RHI, AD, and CTL. Supplementary Table 5. Plasma network module correlations with immunoassay-based IL-6, GFAP, and NFL. Supplementary Table 6. Autopsy subcohort characteristics. Supplementary Table 7. Plasma protein differential abundance between CTE+ and CTE-. Supplementary Table 8. Plasma network module associations with CTE and neuropathology ratings. Supplementary Table 9. Plasma network module associations with global cognition in RHI. Supplementary Table 10. Plasma protein differential correlations with global cognition in RHI [file 13024_2025_860_MOESM1_ESM.pdf]

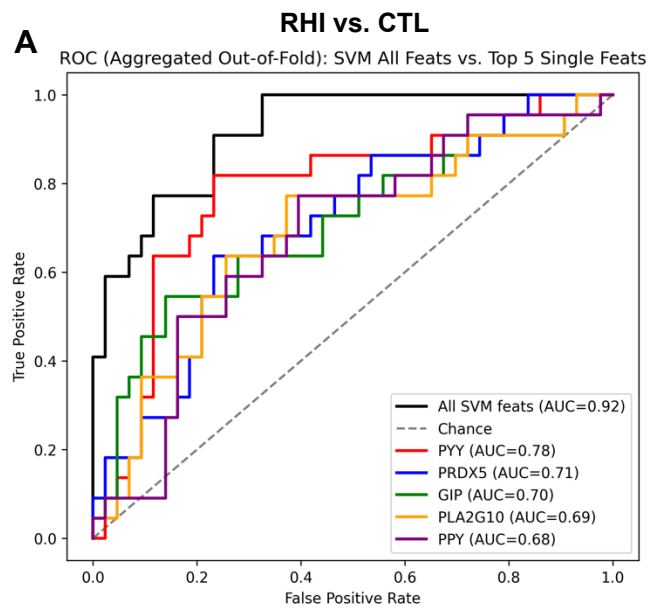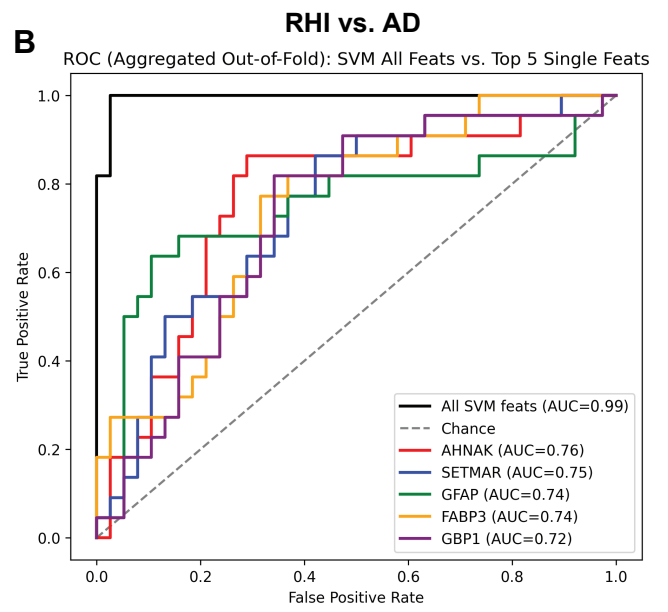

Supplement: Supplementary file 2 — Additional file 2: Supplementary Figure 1. RHI-related proteomic increases are more pronounced in lower-abundance proteins. Olink proteins were binned by absolute concentration in plasma based on data from the Human Protein Atlas. A) Log2 fold-changeof plasma protein abundance in RHI vs. CTL, stratified by protein abundance groups. B) Log2 fold-change of plasma protein abundance in RHI vs. AD, stratified by protein abundance groups. C) Percentage of differentially elevated proteinsin RHI vs. CTL and RHI vs. AD across protein abundance groups. ***p<0.001. [file 13024_2025_860_MOESM2_ESM.pdf]

**A**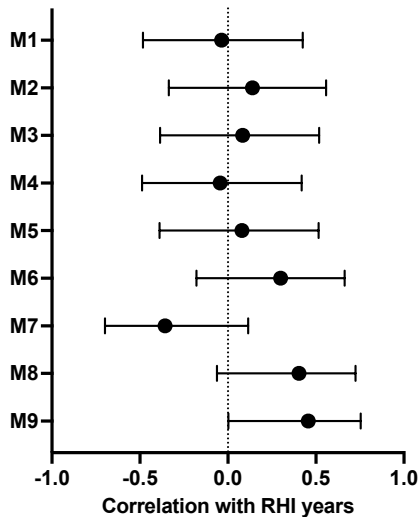**B**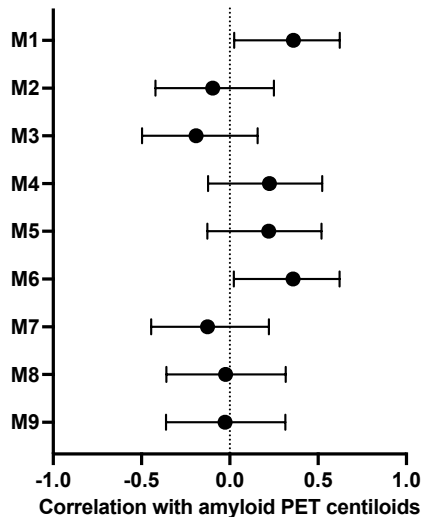

Supplement: Supplementary file 6 — Additional file 6: Supplementary Figure 5. Cell-type enrichment of plasma protein co-expression network modules via web-based cell-specific enrichment analysis. The top 20 general cell types for each module are rank-ordered left to right by the most significant human tissue-cell-type. The red dotted line represents Bonferroni significance threshold across the 1,355 tissue-cell types tested. Negative log10 combined p-values for enrichment of tissue-cell-types are represented along the y-axis. [file 13024_2025_860_MOESM6_ESM.pdf]

**A**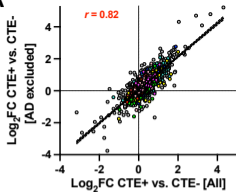**B**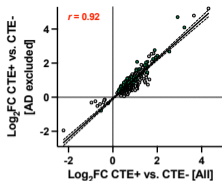

- $p < .05$  both axes (N=72)
- $p < .05$  x-axis only (N=228)

**C**

CTL vs. CTE+ vs. AD

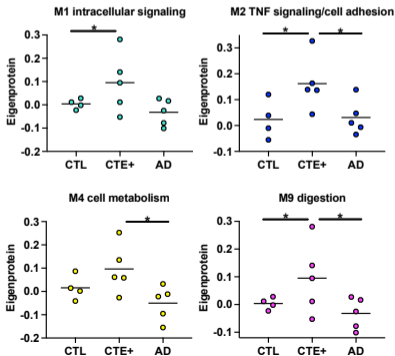

Supplement: Supplementary file 7 — Additional file 7: Supplementary Figure 6. A) Forest plot displaying biweight midcorrelations and 95% confidence intervals for module eigenprotein associations with years of RHI exposure in RHI cases. B) Forest plot displaying biweight midcorrelations and 95% confidence intervals for module eigenprotein associations with amyloid PET centiloids in AD cases [file 13024_2025_860_MOESM7_ESM.pdf]

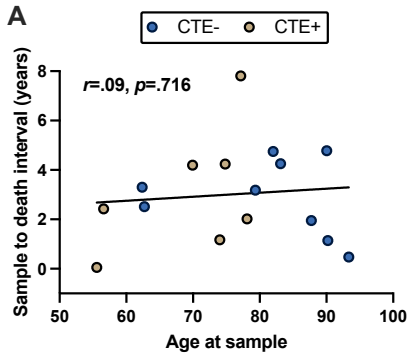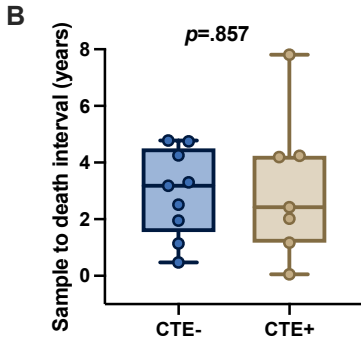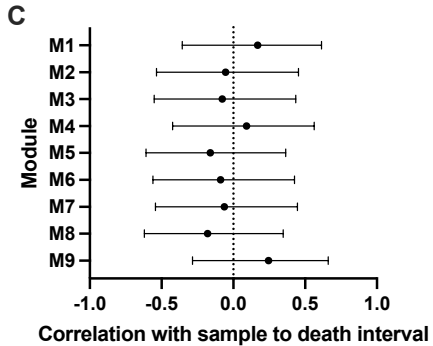

Supplement: Supplementary file 8 — Additional file 8: Supplementary Figure 7. A) Log2-fold changes for all 2,779 plasma proteins across all CTE+ casesvs. CTE-casescompared to log2-fold changes in cases excluded for AD co-pathology. Proteins are color-coded by protein co-expression module assignment. B) Same comparisons as panel A, restricted to the 300 plasma proteins with differential abundance in all CTE+ cases vs. CTE- cases. Proteins are color-coded based on statistical significancein both the all-inclusive and AD-excluded comparisonsor only the all-inclusive comparison. C) Eigenprotein levels in autopsy-confirmed CTL without AD, CTE+ cases without AD, and AD cases without CTEcases for M1, M2, M4, and M9. *p<0.05. [file 13024_2025_860_MOESM8_ESM.pdf]

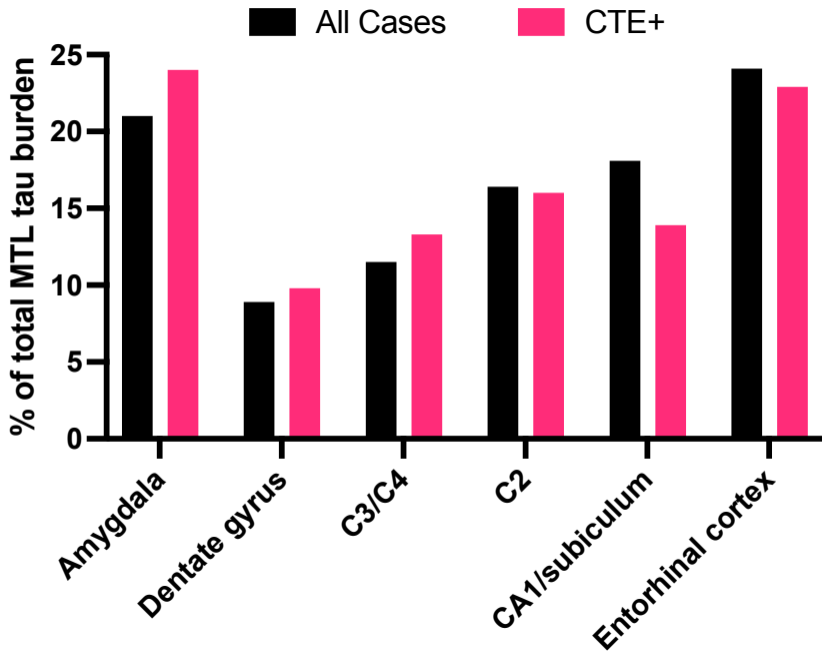

Supplement: Supplementary file 9 — Additional file 9: Supplementary Figure 8. A) Correlation between age at sample collection and the interval between sample collection and death in autopsy cases. Data points are color-coded by CTE+ and CTE-status. B) Box plot of sample to death interval years by CTE status. Box plots represent the median and 25 th and 75 th percentiles, and box hinges represent the interquartile range of the two middle quartiles within a group. Min and max data points define the extent of whiskers. C) Forest plot displaying biweight midcorrelations and 95% confidence intervals for module eigenprotein associations with sample to death intervals [file 13024_2025_860_MOESM9_ESM.pdf]
