# Supplementary material for: Interrogating the plasma proteome of repetitive head impact exposure and chronic traumatic encephalopathy
Source: Mol Neurodegener. 2025 Jun 16;20:71. doi: 10.1186/s13024-025-00860-x (PMC12168330; doi:10.1186/s13024-025-00860-x)

M7 black module: cell morphogenesis

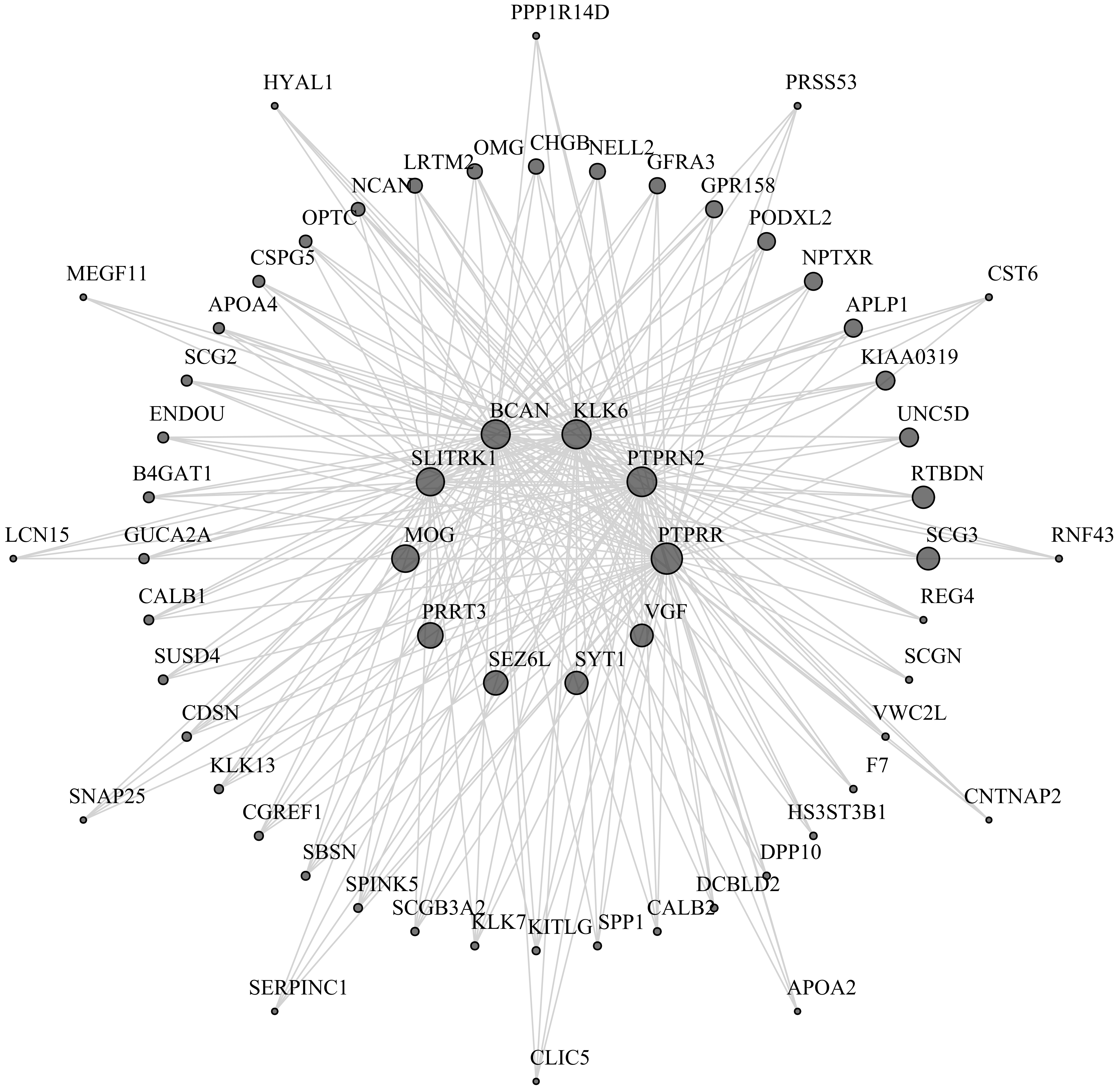

M3 brown module: neurodevelopment/integrin

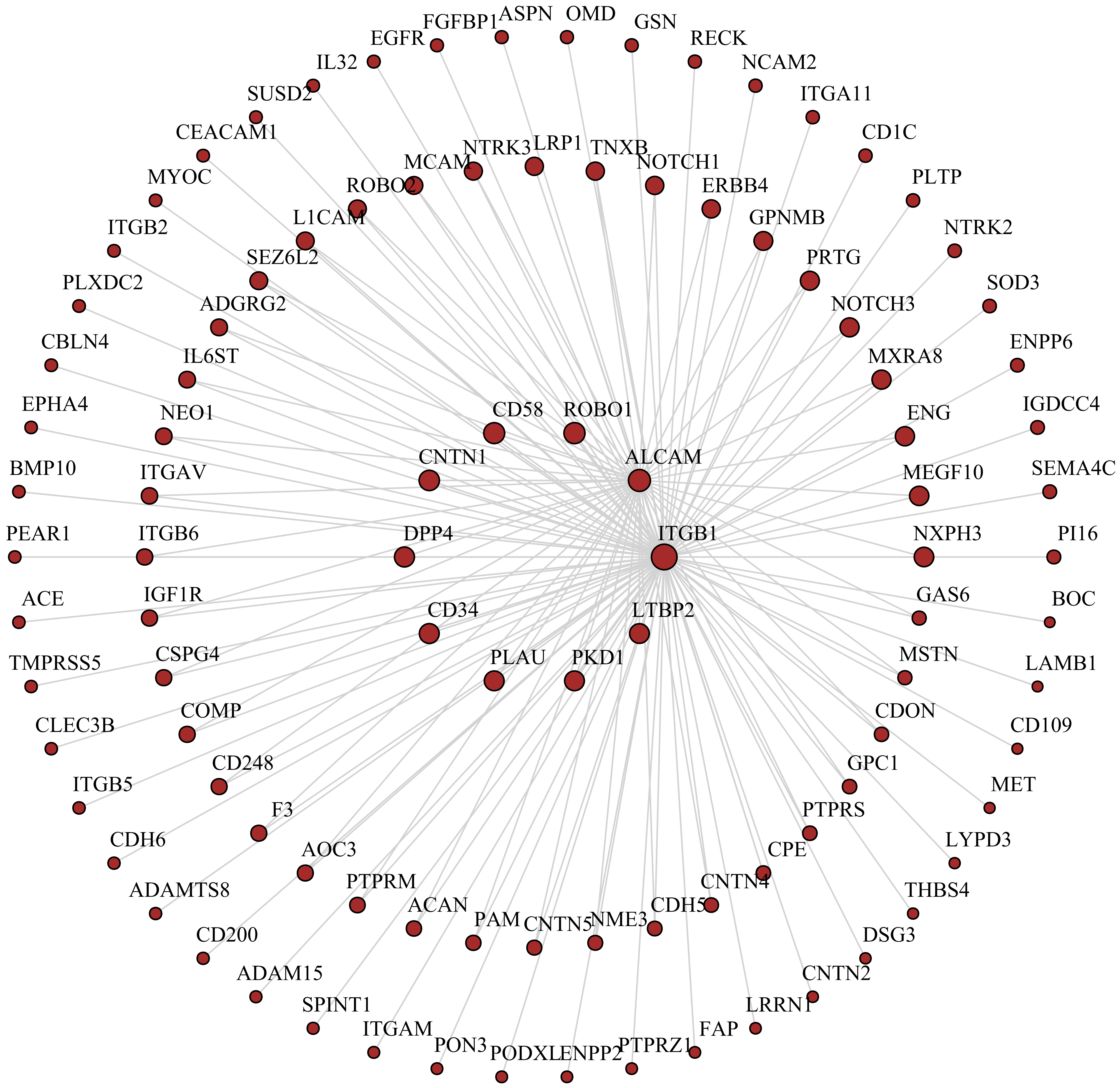

M9 magenta module: digestion

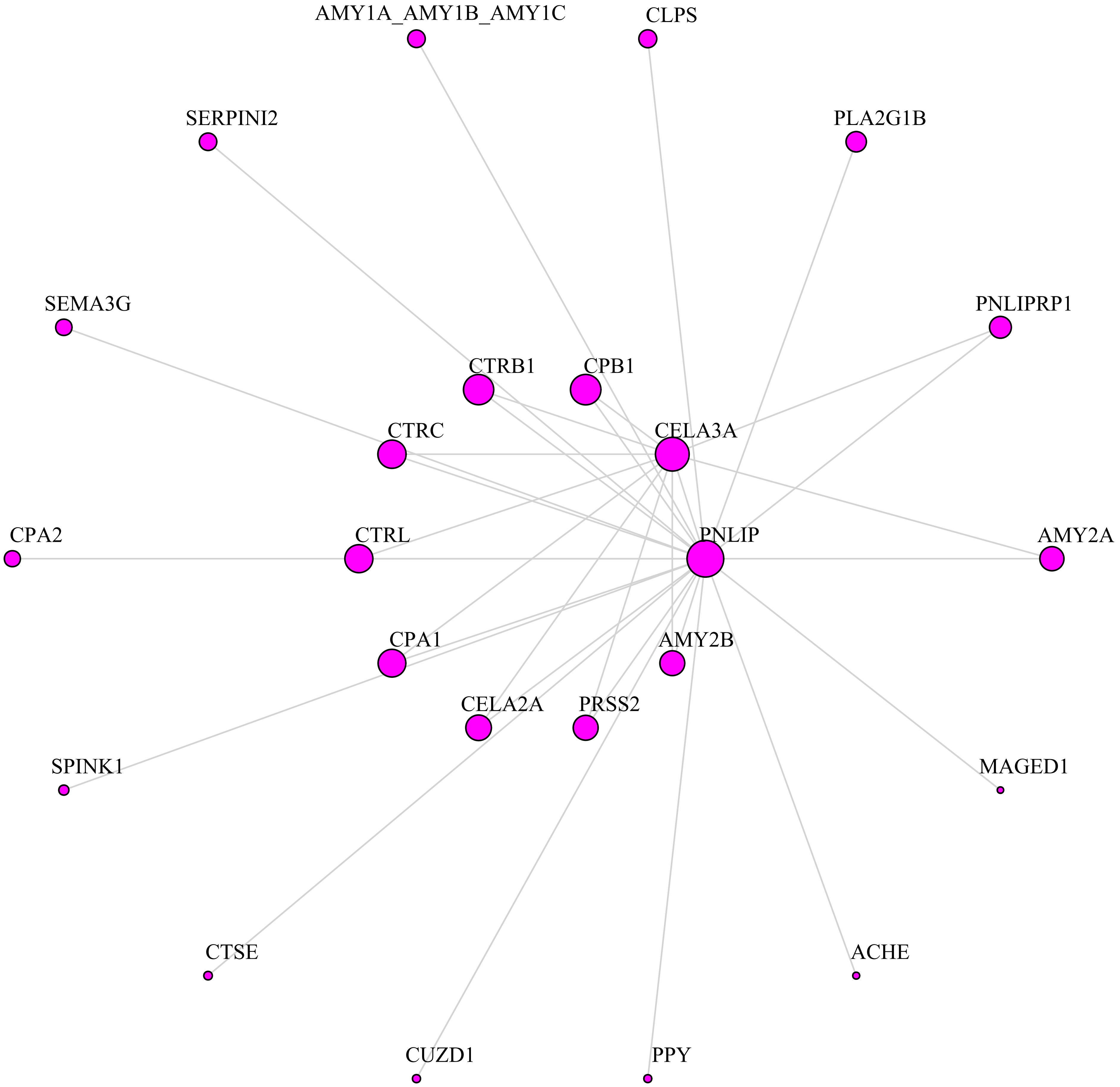

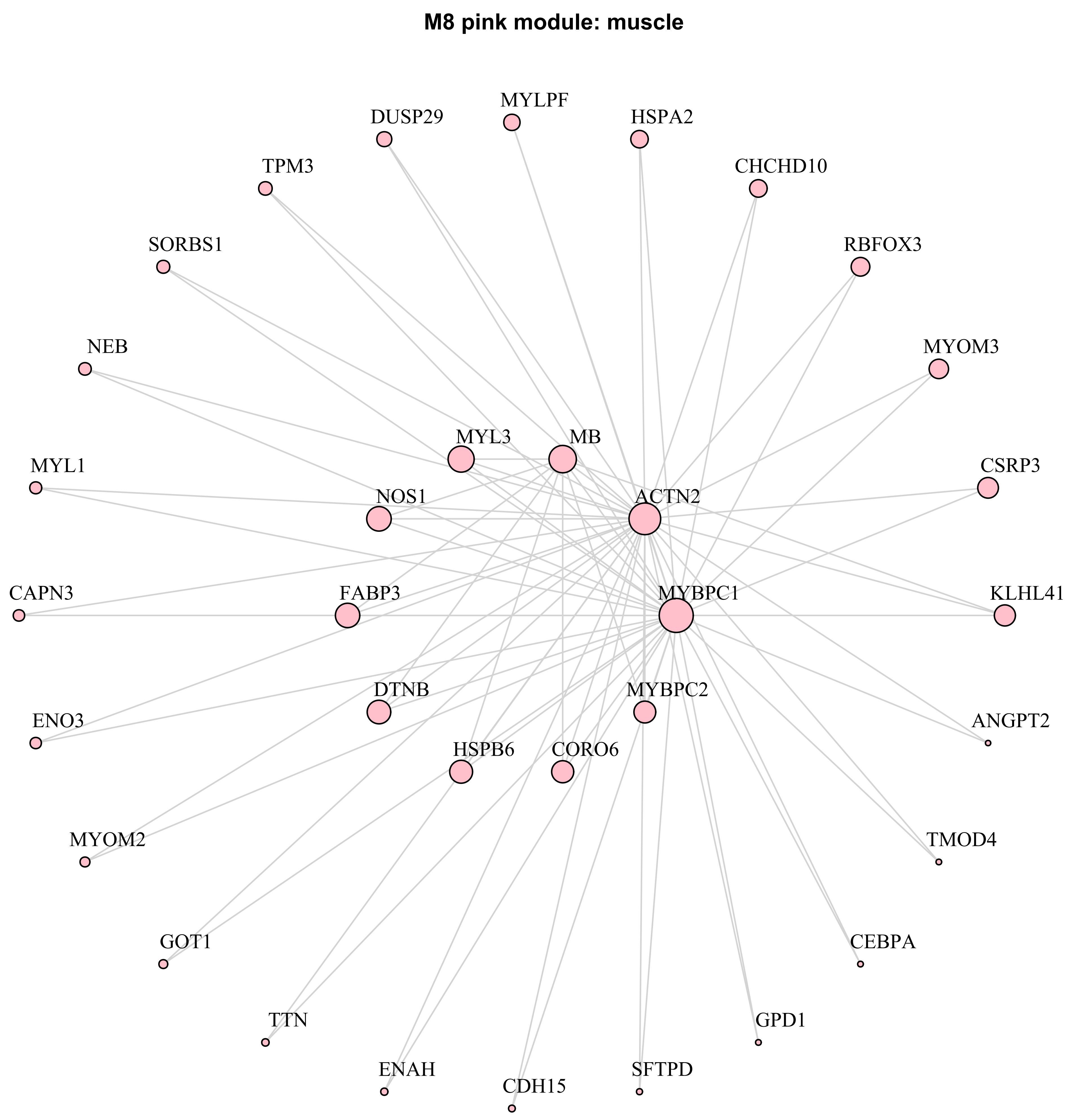
M8 pink module: muscle
DUSP29
MYLPF
HSPA2
TPM3
CHCHD10
SORBS1
RBFOX3
NEB
MYOM3
MYL3
MB
MYL1
CSRP3
NOS1
ACTN2
CAPN3
FABP3
MYBPC1
KLHL41
DTNB
MYBPC2
ENO3
ANGPT2
HSPB6
CORO6
MYOM2
TMOD4
GOT1
CEBPA
TTN
GPD1
ENAH
SFTPD
CDH15

M4 yellow module: cell metabolism

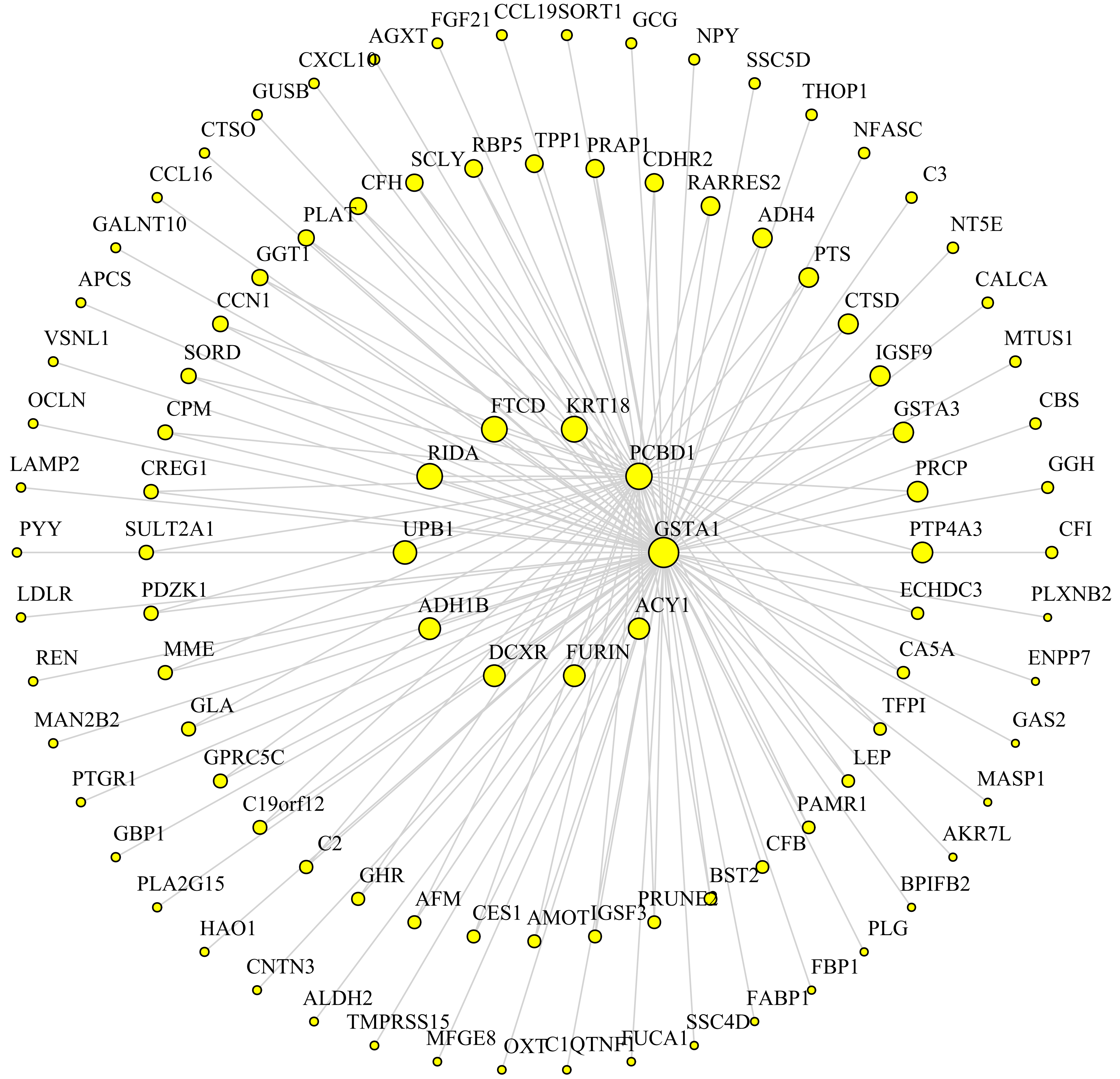

M2 blue module: TNF-signaling/cell adhesion

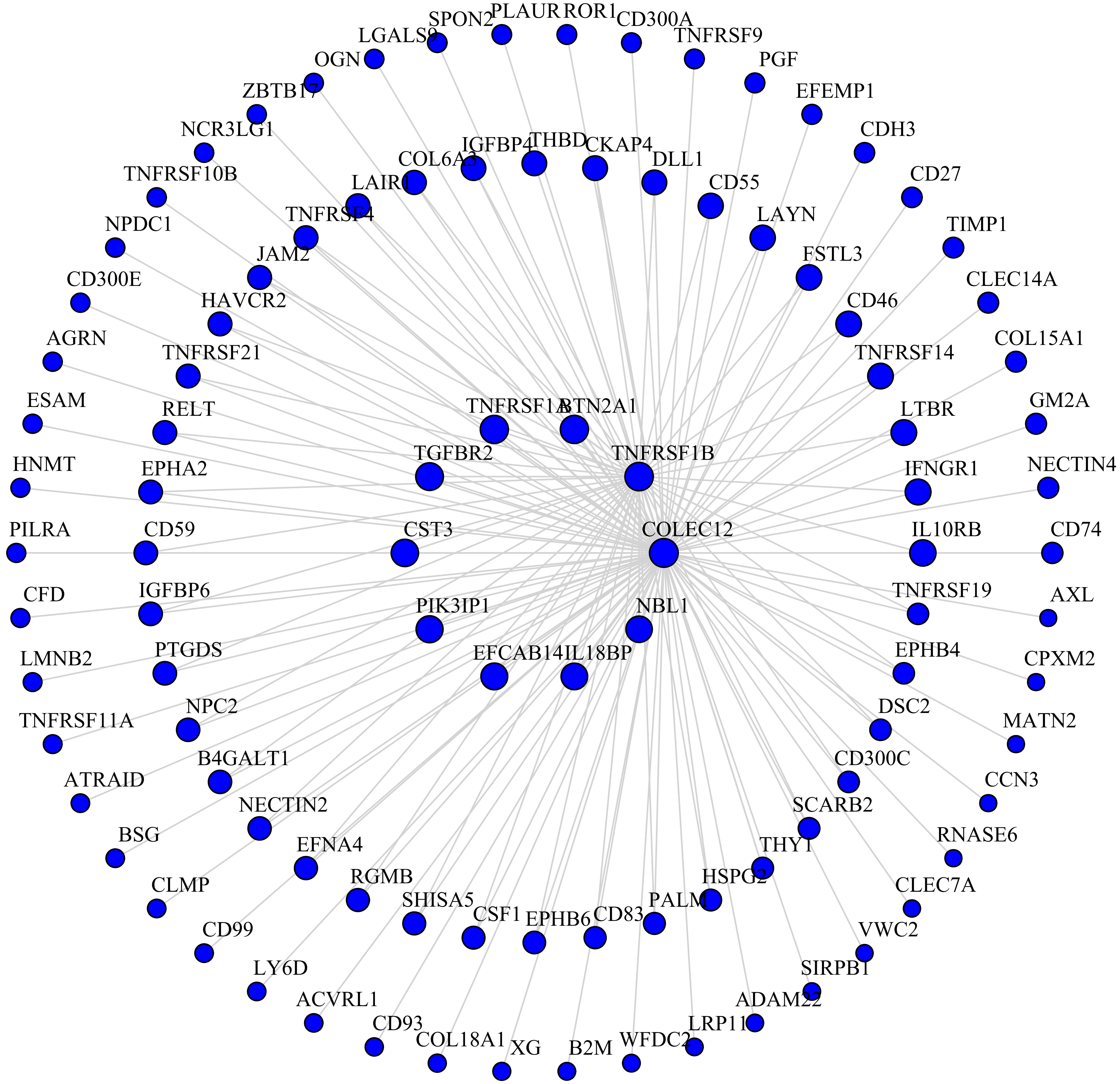

M5 green module: chemotaxis

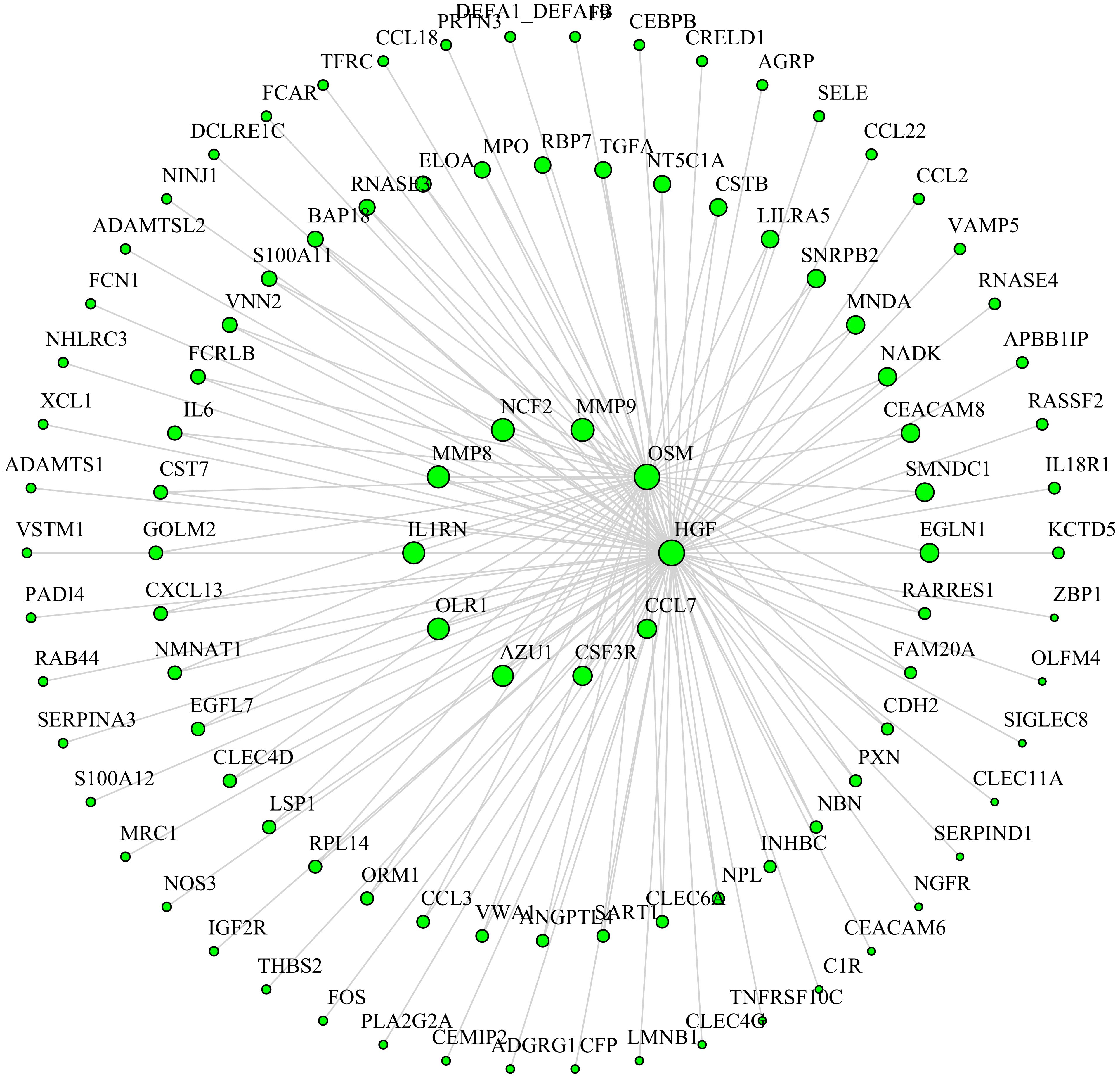

M6 red module: cellular detoxification

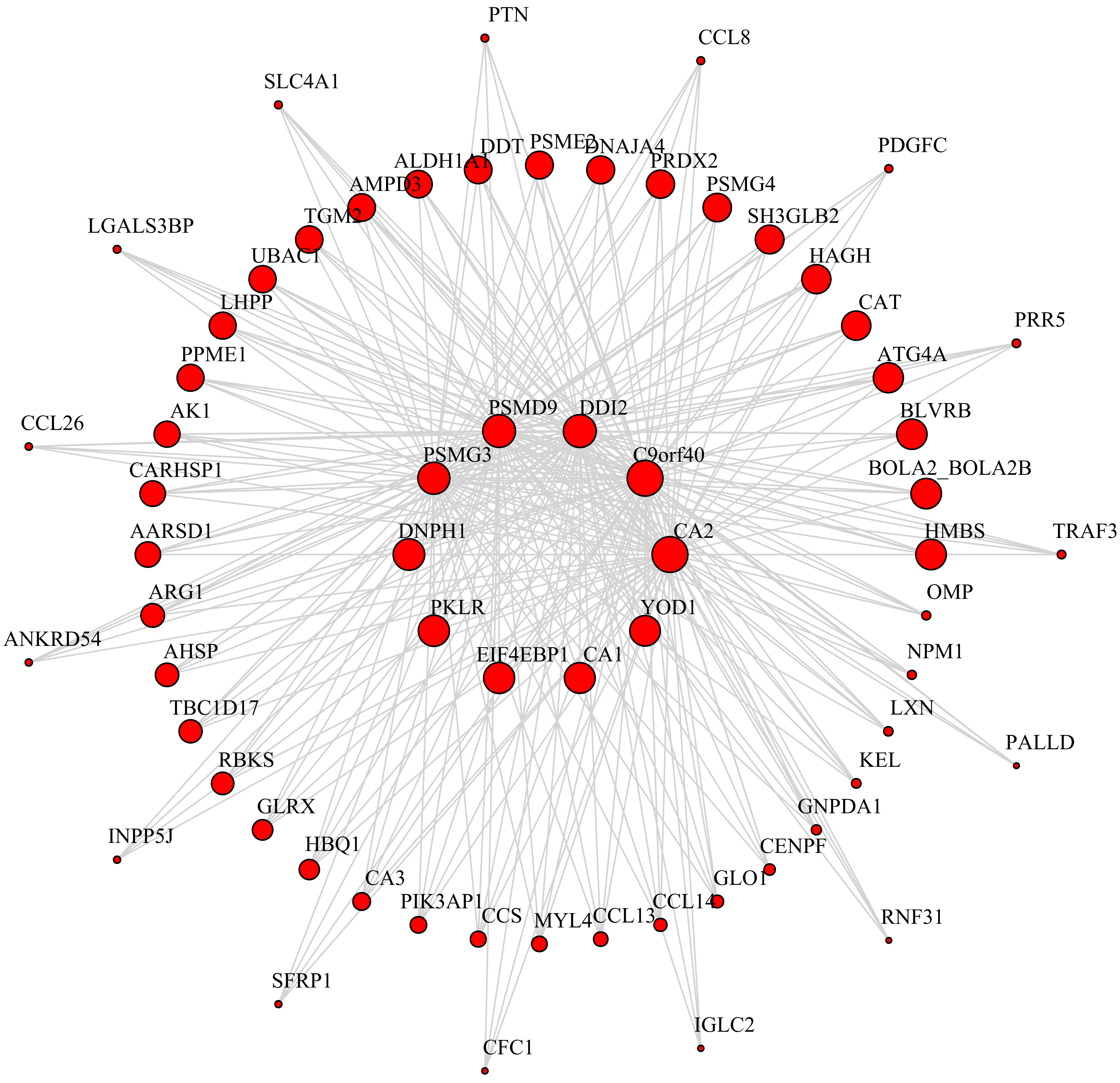

M1 turquoise module: intracellular signaling

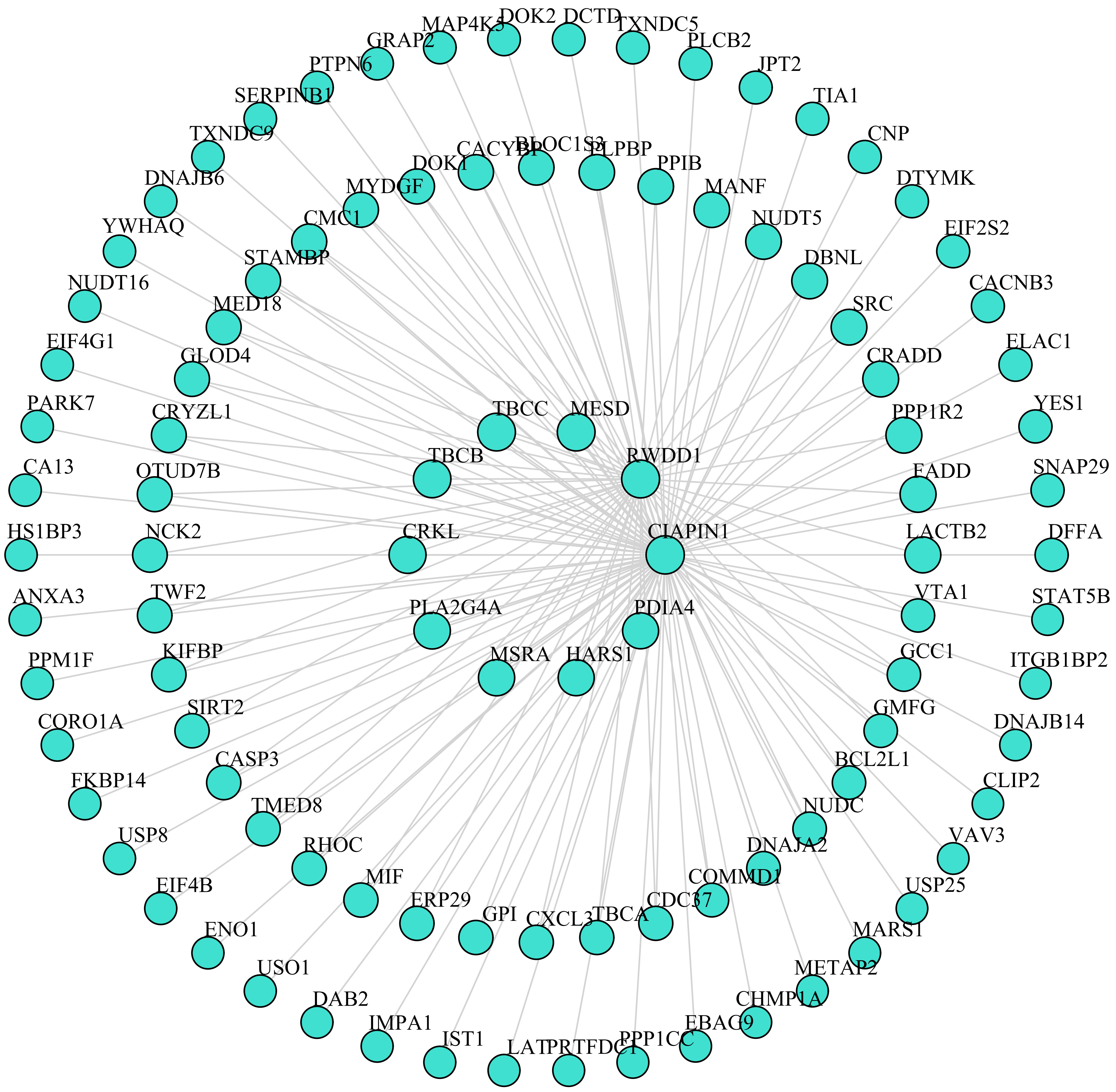

Supplement: Supplementary file 3 — Additional file 3: Supplementary Figure 2. Recursive feature elimination with cross validationselects optimal subsets of proteins that discriminate RHI from CTL and AD. Receiver-operating characteristic curves are plotted for the combined panel of RFECV selected proteins as well as the top 5 proteins, defined by area under the curve, for discriminating A) RHI vs. CTL and B) RHI vs. AD [file 13024_2025_860_MOESM3_ESM.pdf]
