# Supplementary material for: Interrogating the plasma proteome of repetitive head impact exposure and chronic traumatic encephalopathy
Source: Mol Neurodegener. 2025 Jun 16;20:71. doi: 10.1186/s13024-025-00860-x (PMC12168330; doi:10.1186/s13024-025-00860-x)
Supplement: Supplementary file 4 — Additional file 4: Supplementary Figure 3. Plasma network module protein graphs. The size of each circle indicates the relative eigenprotein correlation valuein each network module. Those proteins with the largest kME are considered “hub” proteins within the module, and explain the largest variance in module expression. The top 150 proteins by kME for each module are shown [file 13024_2025_860_MOESM4_ESM.pdf]

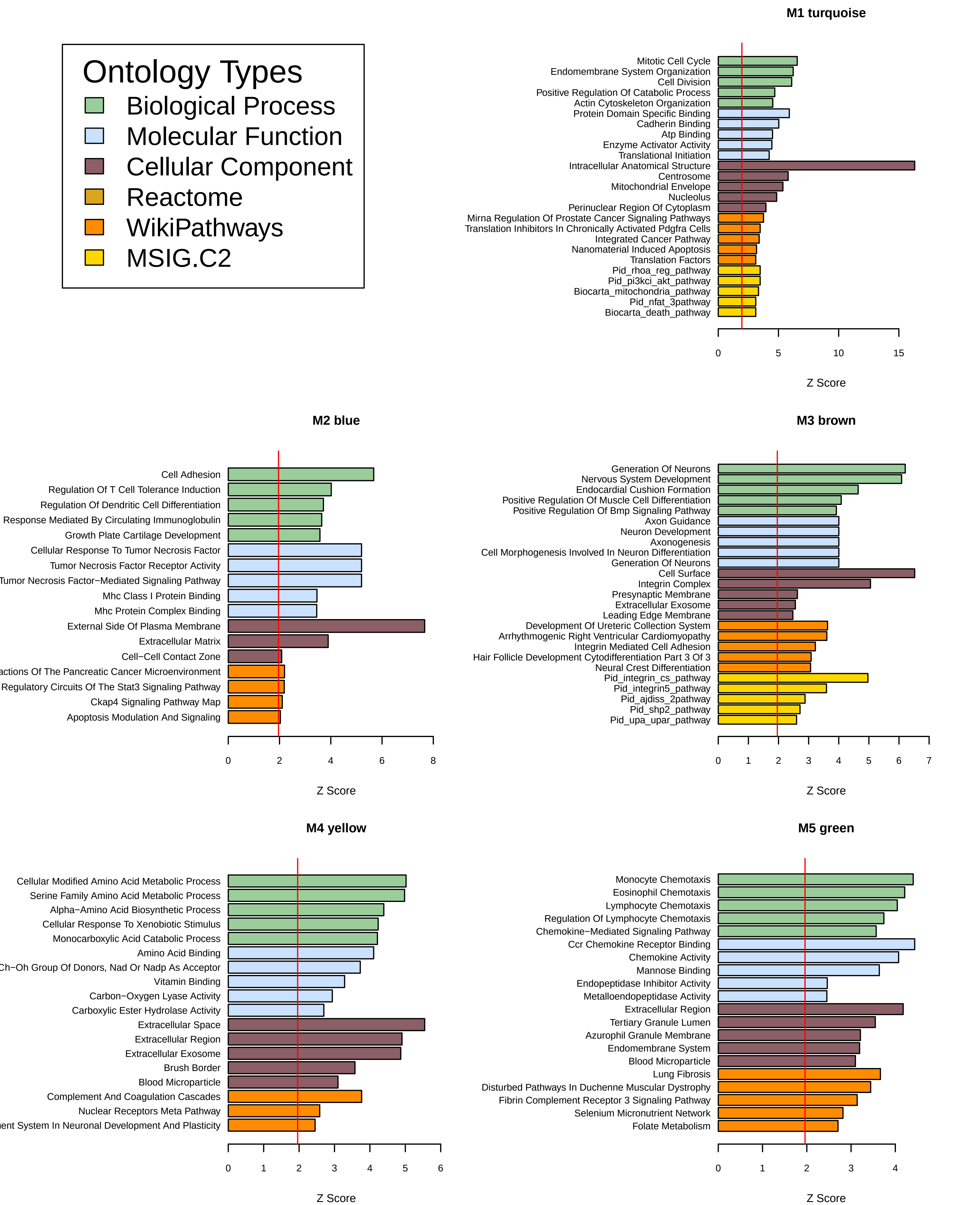
Ontology Types
Biological Process
Molecular Function
Cellular Component
Reactome
WikiPathways
MSIG.C2
M1 turquoise
Mitotic Cell Cycle
Endomembrane System Organization
Cell Division
Positive Regulation Of Catabolic Process
Actin Cytoskeleton Organization
Protein Domain Specific Binding
Cadherin Binding
Atp Binding
Enzyme Activator Activity
Translational Initiation
Intracellular Anatomical Structure
Centrosome
Mitochondrial Envelope
Nucleolus
Perinuclear Region Of Cytoplasm
Mirna Regulation Of Prostate Cancer Signaling Pathways
Translation Inhibitors In Chronically Activated Pdgfra Cells
Integrated Cancer Pathway
Nanomaterial Induced Apoptosis
Translation Factors
Pid_rhoa_reg_pathway
Pid_pi3kci_akt_pathway
Biocarta_mitochondria_pathway
Pid_nfat_3pathway
Biocarta_death_pathway
0
5
10
15
Z Score
M2 blue
Cell Adhesion
Regulation Of T Cell Tolerance Induction
Regulation Of Dendritic Cell Differentiation
Response Mediated By Circulating Immunoglobulin
Growth Plate Cartilage Development
Cellular Response To Tumor Necrosis Factor
Tumor Necrosis Factor Receptor Activity
Tumor Necrosis Factor−Mediated Signaling Pathway
Mhc Class I Protein Binding
Mhc Protein Complex Binding
External Side Of Plasma Membrane
Extracellular Matrix
Cell−Cell Contact Zone
actions Of The Pancreatic Cancer Microenvironment
Regulatory Circuits Of The Stat3 Signaling Pathway
Ckap4 Signaling Pathway Map
Apoptosis Modulation And Signaling
0
2
4
6
8
Z Score
M3 brown
Generation Of Neurons
Nervous System Development
Endocardial Cushion Formation
Positive Regulation Of Muscle Cell Differentiation
Positive Regulation Of Bmp Signaling Pathway
Axon Guidance
Neuron Development
Axonogenesis
Cell Morphogenesis Involved In Neuron Differentiation
Generation Of Neurons
Cell Surface
Integrin Complex
Presynaptic Membrane
Extracellular Exosome
Leading Edge Membrane
Development Of Ureteric Collection System
Arrhythmogenic Right Ventricular Cardiomyopathy
Integrin Mediated Cell Adhesion
Hair Follicle Development Cytodifferentiation Part 3 Of 3
Neural Crest Differentiation
Pid_integrin_cs_pathway
Pid_integrin5_pathway
Pid_ajdiss_2pathway
Pid_shp2_pathway
Pid_upa_upar_pathway
0
1
2
3
4
5
6
7
Z Score
M4 yellow
Cellular Modified Amino Acid Metabolic Process
Serine Family Amino Acid Metabolic Process
Alpha−Amino Acid Biosynthetic Process
Cellular Response To Xenobiotic Stimulus
Monocarboxylic Acid Catabolic Process
Amino Acid Binding
Ch−Oh Group Of Donors, Nad Or Nadp As Acceptor
Vitamin Binding
Carbon−Oxygen Lyase Activity
Carboxylic Ester Hydrolase Activity
Extracellular Space
Extracellular Region
Extracellular Exosome
Brush Border
Blood Microparticle
Complement And Coagulation Cascades
Nuclear Receptors Meta Pathway
ent System In Neuronal Development And Plasticity
0
1
2
3
4
5
6
Z Score
M5 green
Monocyte Chemotaxis
Eosinophil Chemotaxis
Lymphocyte Chemotaxis
Regulation Of Lymphocyte Chemotaxis
Chemokine−Mediated Signaling Pathway
Ccr Chemokine Receptor Binding
Chemokine Activity
Mannose Binding
Endopeptidase Inhibitor Activity
Metalloendopeptidase Activity
Extracellular Region
Tertiary Granule Lumen
Azurophil Granule Membrane
Endomembrane System
Blood Microparticle
Lung Fibrosis
Disturbed Pathways In Duchenne Muscular Dystrophy
Fibrin Complement Receptor 3 Signaling Pathway
Selenium Micronutrient Network
Folate Metabolism
0
1
2
3
4
Z Score

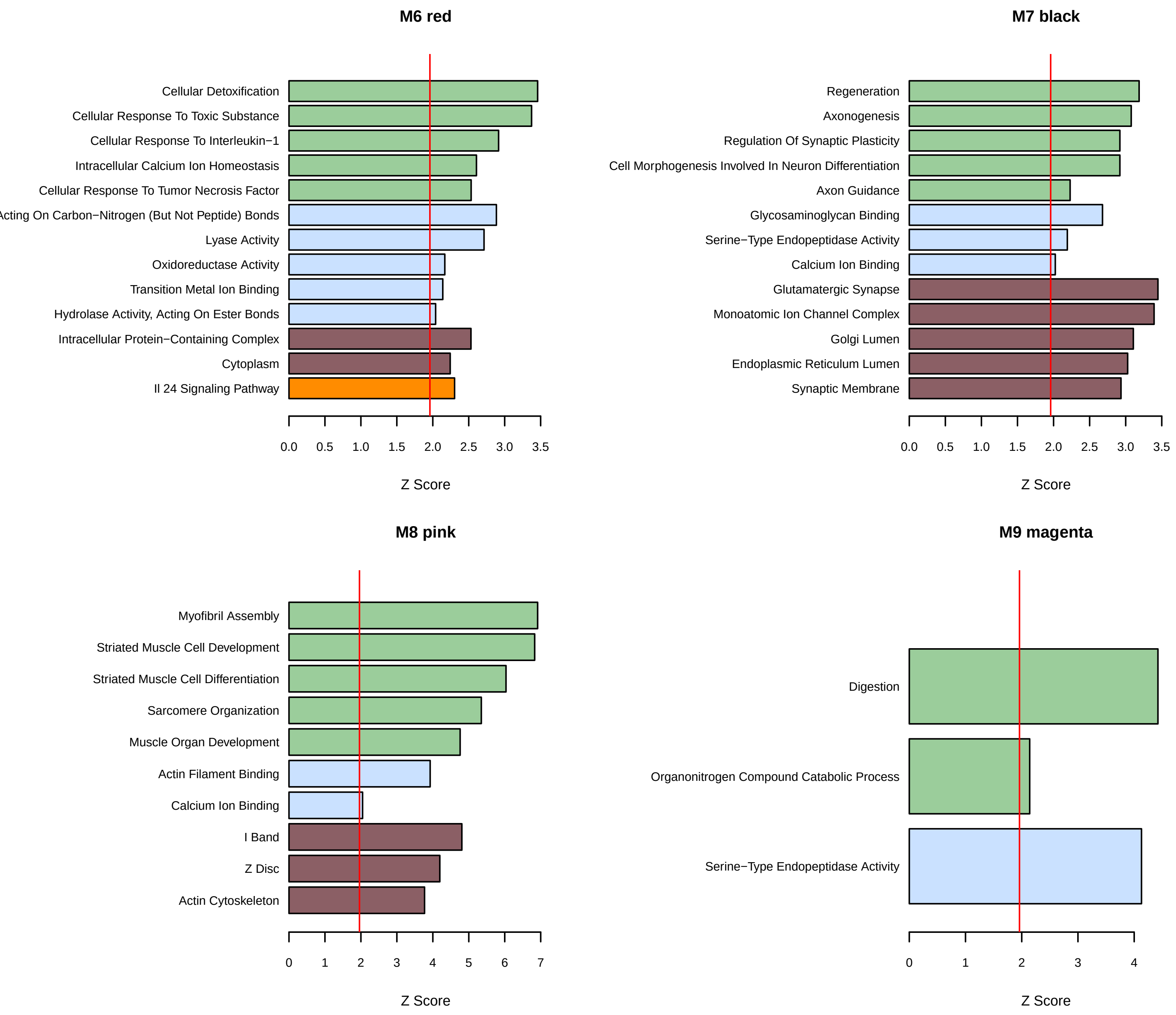

M6 red
Cellular Detoxification
Cellular Response To Toxic Substance
Cellular Response To Interleukin−1
Intracellular Calcium Ion Homeostasis
Cellular Response To Tumor Necrosis Factor
Acting On Carbon−Nitrogen (But Not Peptide) Bonds
Lyase Activity
Oxidoreductase Activity
Transition Metal Ion Binding
Hydrolase Activity, Acting On Ester Bonds
Intracellular Protein−Containing Complex
Cytoplasm
Il 24 Signaling Pathway
0.0 0.5 1.0 1.5 2.0 2.5 3.0 3.5
Z Score
M7 black
Regeneration
Axonogenesis
Regulation Of Synaptic Plasticity
Cell Morphogenesis Involved In Neuron Differentiation
Axon Guidance
Glycosaminoglycan Binding
Serine−Type Endopeptidase Activity
Calcium Ion Binding
Glutamatergic Synapse
Monoatomic Ion Channel Complex
Golgi Lumen
Endoplasmic Reticulum Lumen
Synaptic Membrane
0.0 0.5 1.0 1.5 2.0 2.5 3.0 3.5
Z Score
M8 pink
Myofibril Assembly
Striated Muscle Cell Development
Striated Muscle Cell Differentiation
Sarcomere Organization
Muscle Organ Development
Actin Filament Binding
Calcium Ion Binding
I Band
Z Disc
Actin Cytoskeleton
0 1 2 3 4 5 6 7
Z Score
M9 magenta
Digestion
Organonitrogen Compound Catabolic Process
Serine−Type Endopeptidase Activity
0 1 2 3 4
Z Score
