# Supplementary material for: Interrogating the plasma proteome of repetitive head impact exposure and chronic traumatic encephalopathy
Source: Mol Neurodegener. 2025 Jun 16;20:71. doi: 10.1186/s13024-025-00860-x (PMC12168330; doi:10.1186/s13024-025-00860-x)
Supplement: Supplementary file 5 — Additional file 5: Supplementary Figure 4. Biological enrichment of plasma protein co-expression network modules. Gene ontologyand pathway analysis was performed to ascertain the principal biology represented by the constituent proteins in each module. Enrichment for a given ontology is shown by z score, transformed from a Fisher’s exact test. [file 13024_2025_860_MOESM5_ESM.pdf]

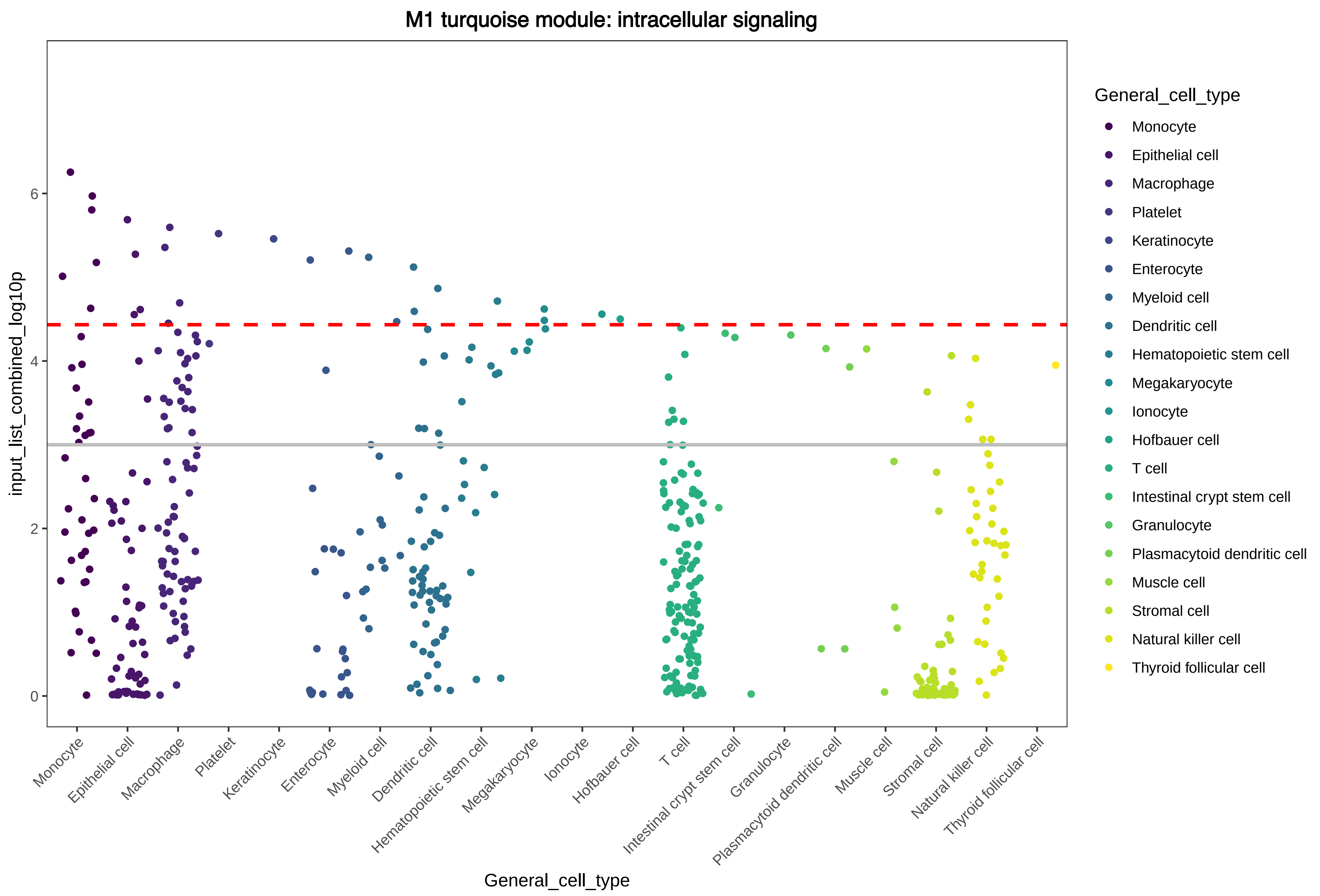

M1 turquoise module: intracellular signaling
input_list_combined_log10p
0
2
4
6
General_cell_type
Monocyte
Epithelial cell
Macrophage
Platelet
Keratinocyte
Enterocyte
Myeloid cell
Dendritic cell
Hematopoietic stem cell
Megakaryocyte
Ionocyte
Hofbauer cell
T cell
Intestinal crypt stem cell
Granulocyte
Plasmacytoid dendritic cell
Muscle cell
Stromal cell
Natural killer cell
Thyroid follicular cell

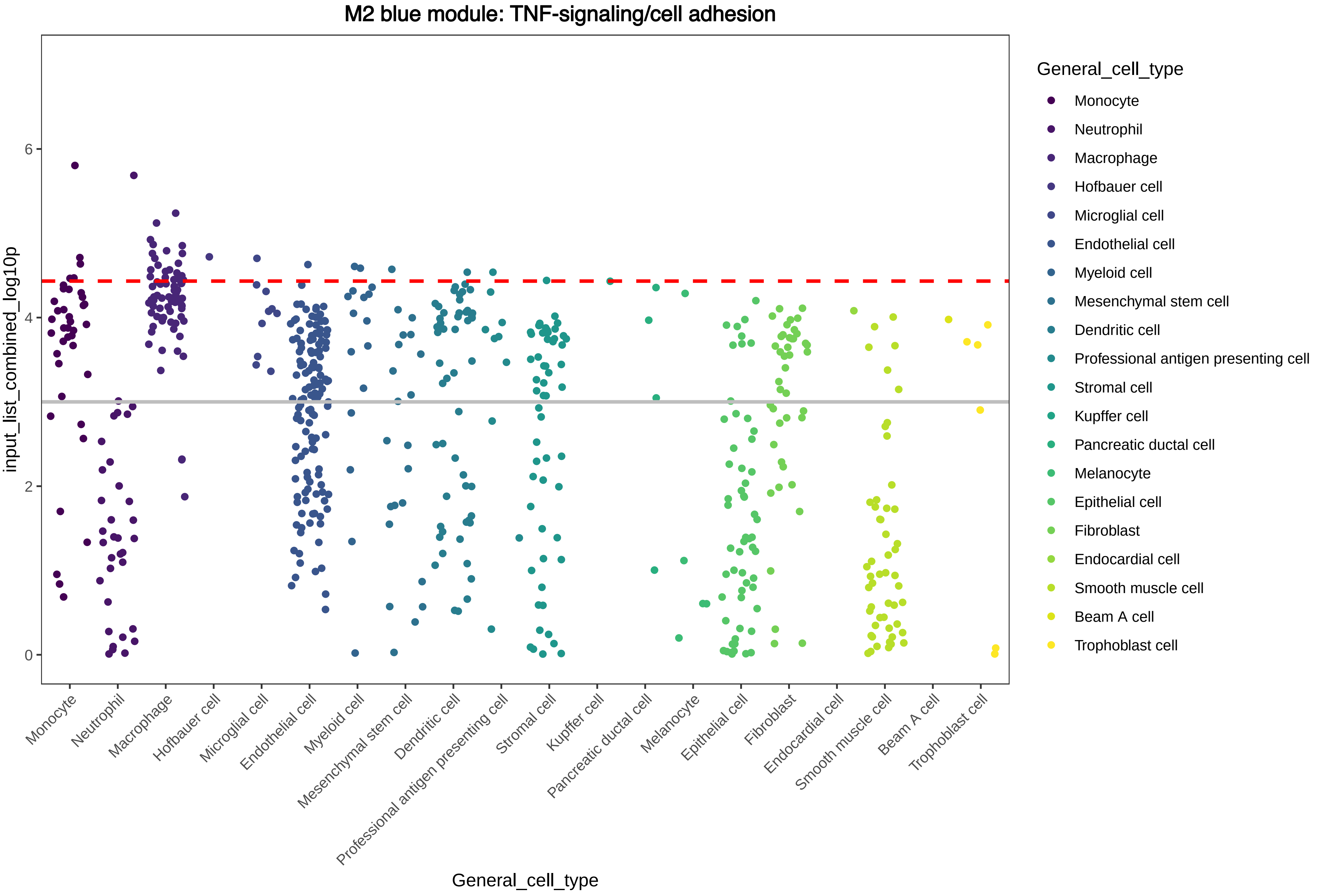

M2 blue module: TNF-signaling/cell adhesion
input_list_combined_log10p
0
2
4
6
General_cell_type
Monocyte
Neutrophil
Macrophage
Hofbauer cell
Microglial cell
Endothelial cell
Myeloid cell
Mesenchymal stem cell
Dendritic cell
Professional antigen presenting cell
Stromal cell
Kupffer cell
Pancreatic ductal cell
Melanocyte
Epithelial cell
Fibroblast
Endocardial cell
Smooth muscle cell
Beam A cell
Trophoblast cell

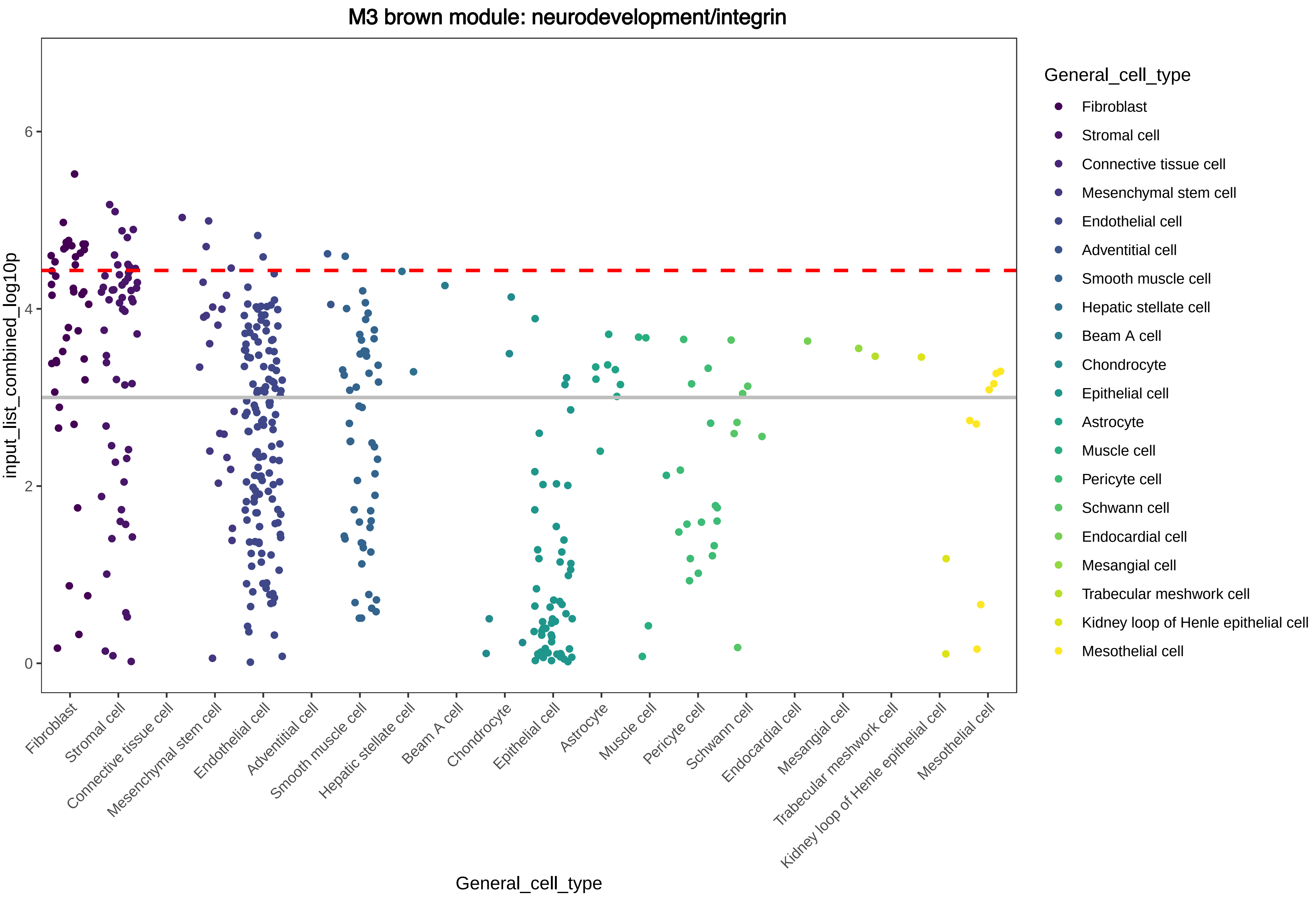

M3 brown module: neurodevelopment/integrin
input_list_combined_log10p
0
2
4
6
General_cell_type
Fibroblast
Stromal cell
Connective tissue cell
Mesenchymal stem cell
Endothelial cell
Adventitial cell
Smooth muscle cell
Hepatic stellate cell
Beam A cell
Chondrocyte
Epithelial cell
Astrocyte
Muscle cell
Pericyte cell
Schwann cell
Endocardial cell
Mesangial cell
Trabecular meshwork cell
Kidney loop of Henle epithelial cell
Mesothelial cell

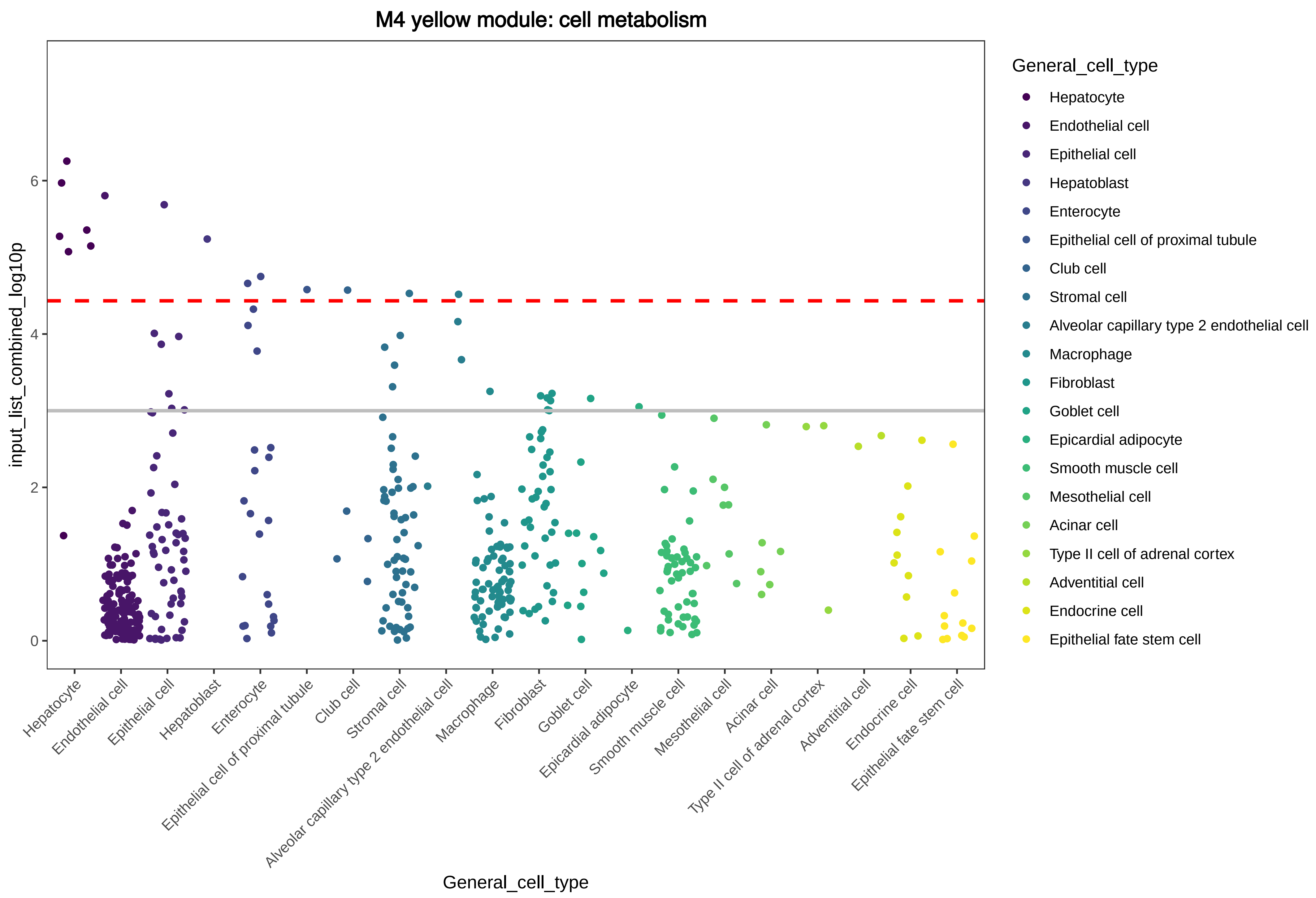

M4 yellow module: cell metabolism
input_list_combined_log10p
0
2
4
6
General_cell_type
Hepatocyte
Endothelial cell
Epithelial cell
Hepatoblast
Enterocyte
Epithelial cell of proximal tubule
Club cell
Stromal cell
Alveolar capillary type 2 endothelial cell
Macrophage
Fibroblast
Goblet cell
Epicardial adipocyte
Smooth muscle cell
Mesothelial cell
Acinar cell
Type II cell of adrenal cortex
Adventitial cell
Endocrine cell
Epithelial fate stem cell

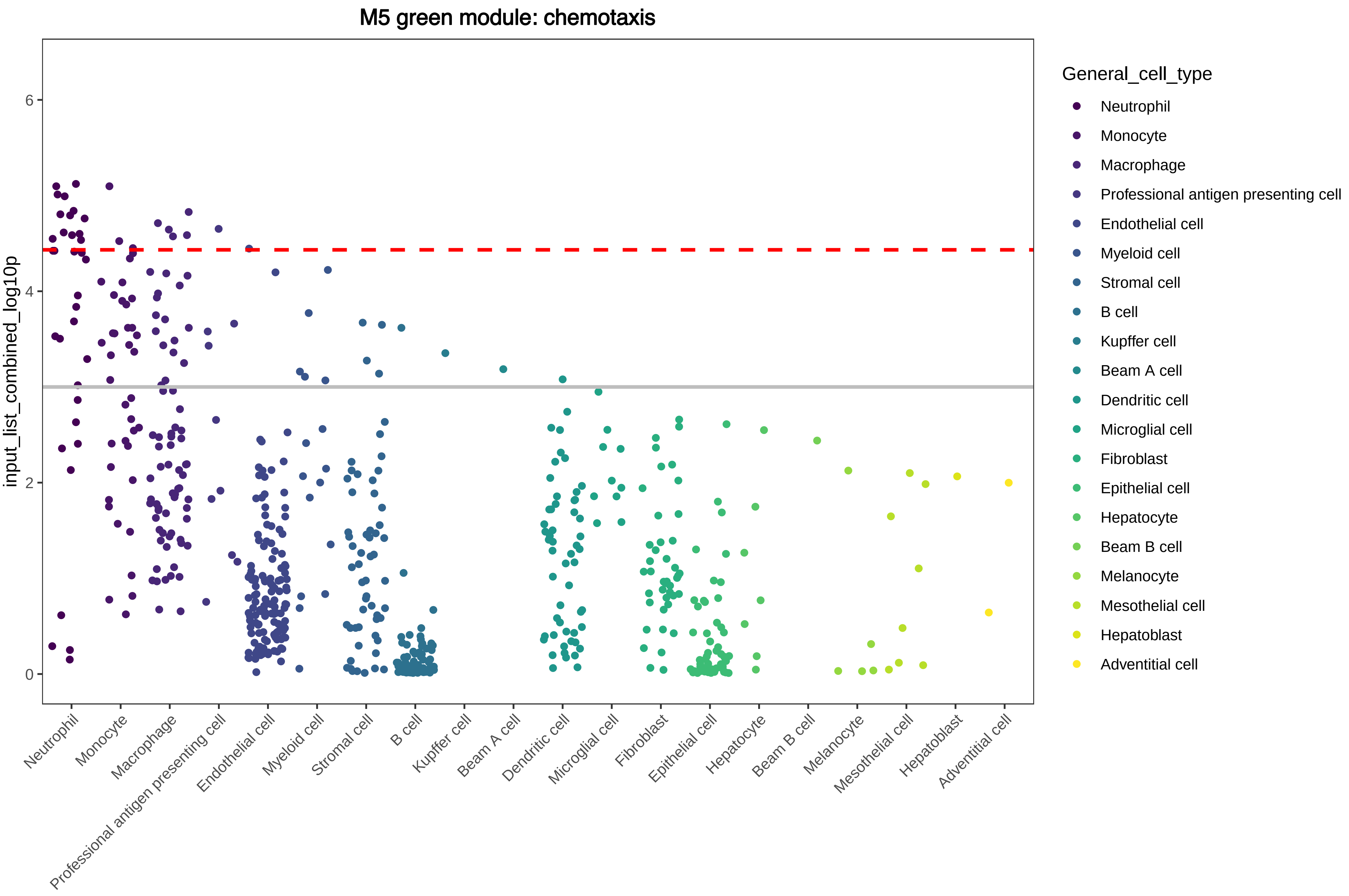
M5 green module: chemotaxis
input_list_combined_log10p
0
2
4
6
General_cell_type
Neutrophil
Monocyte
Macrophage
Professional antigen presenting cell
Endothelial cell
Myeloid cell
Stromal cell
B cell
Kupffer cell
Beam A cell
Dendritic cell
Microglial cell
Fibroblast
Epithelial cell
Hepatocyte
Beam B cell
Melanocyte
Mesothelial cell
Hepatoblast
Adventitial cell

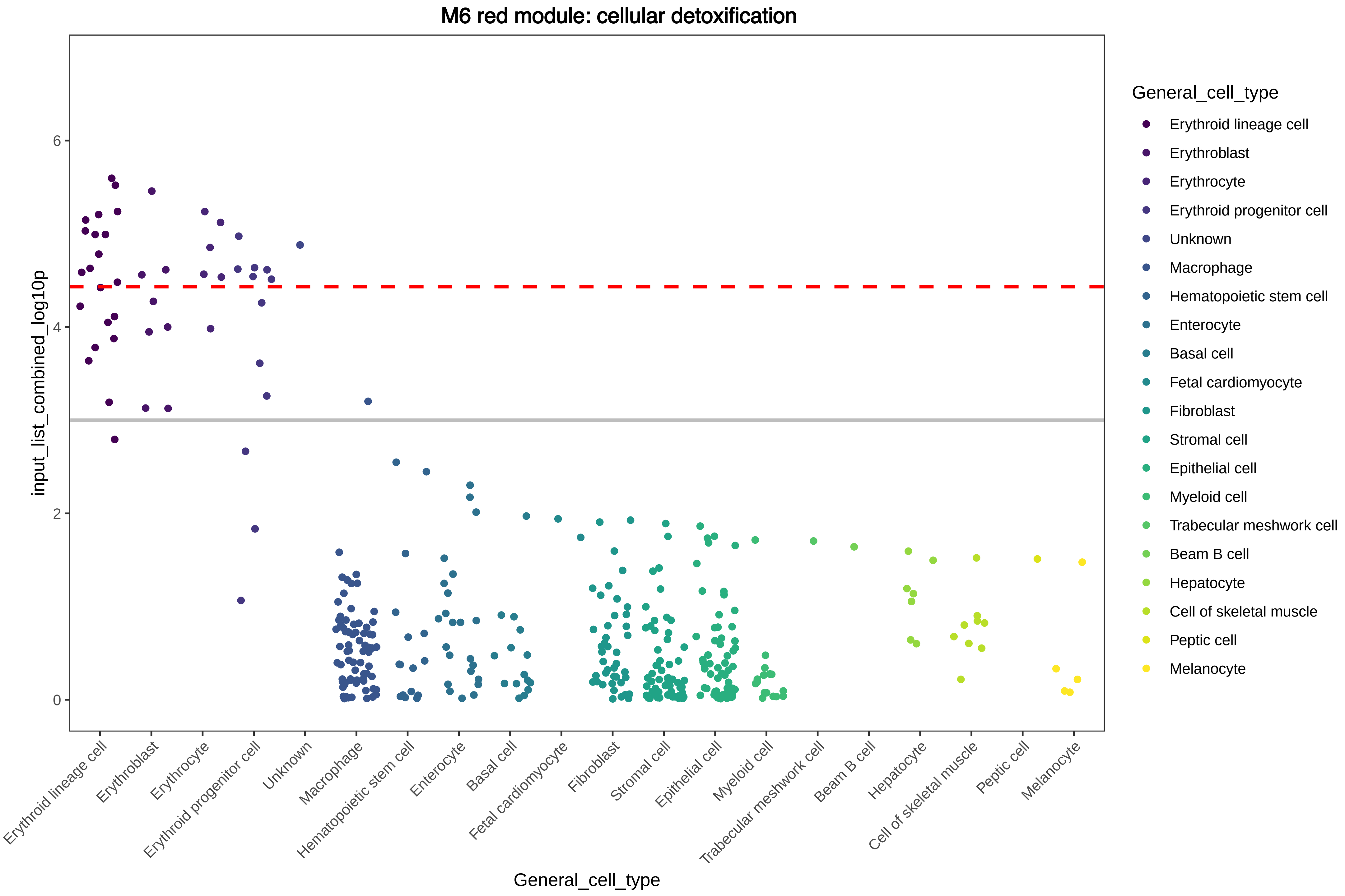

M6 red module: cellular detoxification
input_list_combined_log10p
0
2
4
6
General_cell_type
Erythroid lineage cell
Erythroblast
Erythrocyte
Erythroid progenitor cell
Unknown
Macrophage
Hematopoietic stem cell
Enterocyte
Basal cell
Fetal cardiomyocyte
Fibroblast
Stromal cell
Epithelial cell
Myeloid cell
Trabecular meshwork cell
Beam B cell
Hepatocyte
Cell of skeletal muscle
Peptic cell
Melanocyte
General_cell_type

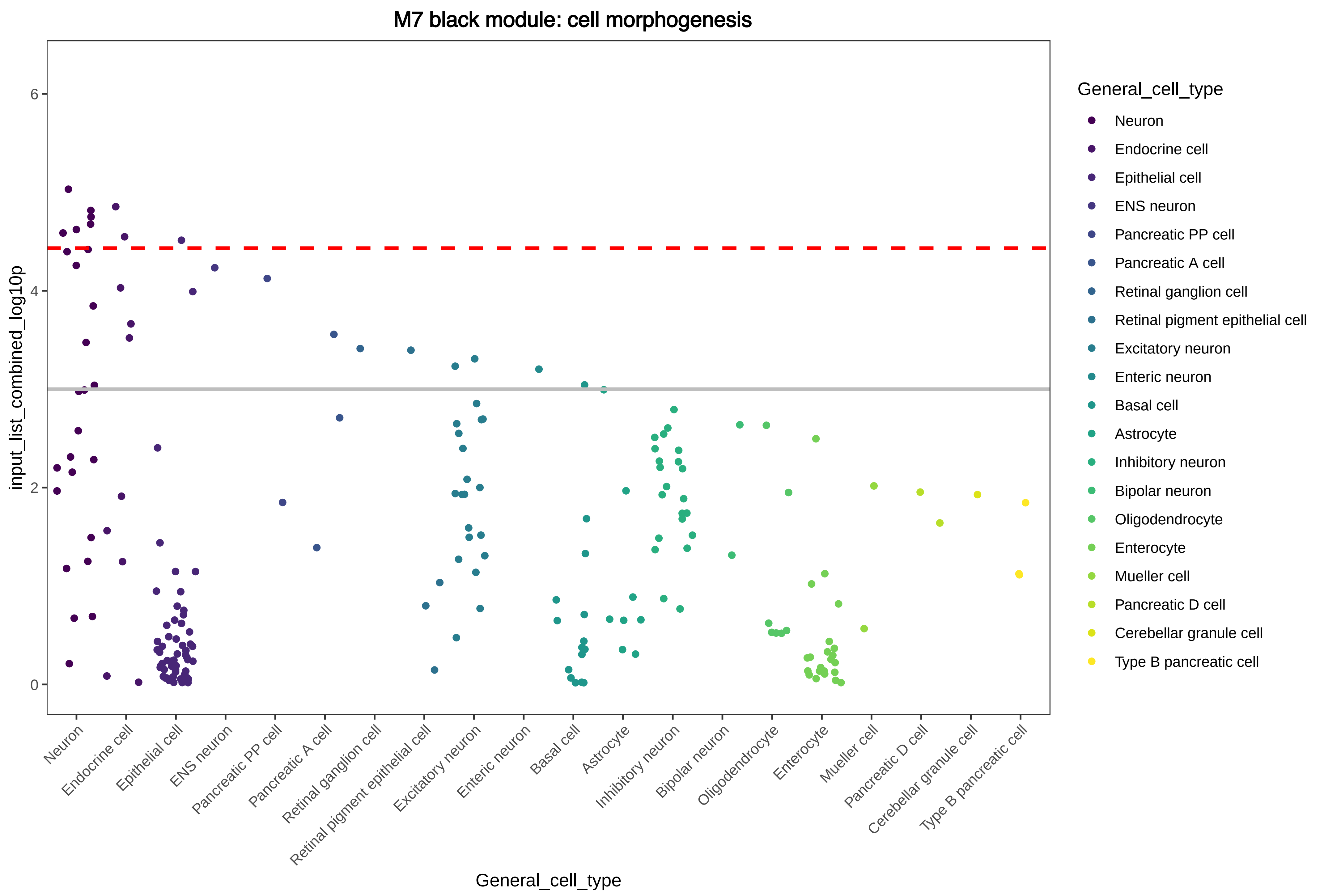

M7 black module: cell morphogenesis
input_list_combined_log10p
0
2
4
6
General_cell_type
Neuron
Endocrine cell
Epithelial cell
ENS neuron
Pancreatic PP cell
Pancreatic A cell
Retinal ganglion cell
Retinal pigment epithelial cell
Excitatory neuron
Enteric neuron
Basal cell
Astrocyte
Inhibitory neuron
Bipolar neuron
Oligodendrocyte
Enterocyte
Mueller cell
Pancreatic D cell
Cerebellar granule cell
Type B pancreatic cell

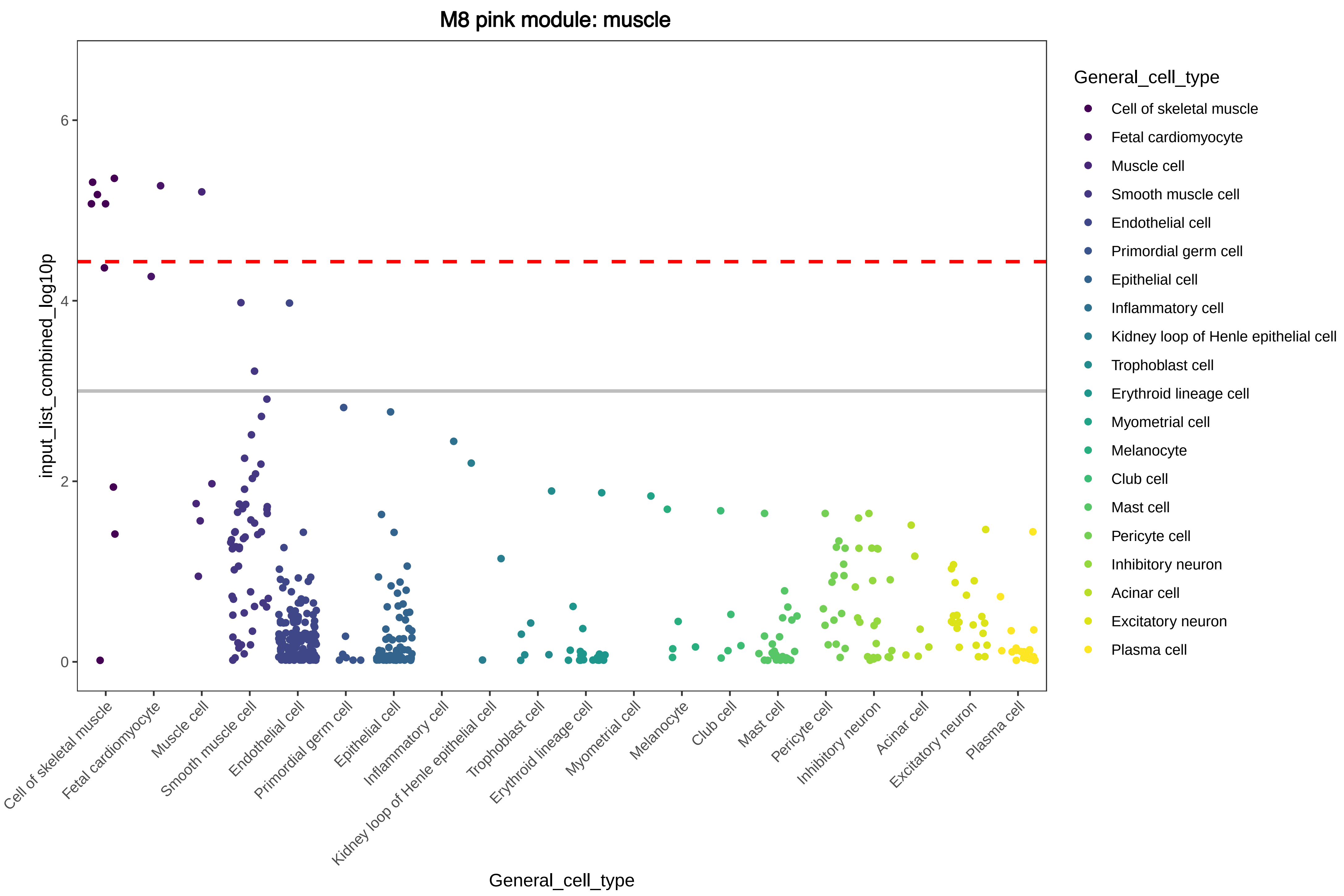

M8 pink module: muscle
input_list_combined_log10p
0
2
4
6
General_cell_type
Cell of skeletal muscle
Fetal cardiomyocyte
Muscle cell
Smooth muscle cell
Endothelial cell
Primordial germ cell
Epithelial cell
Inflammatory cell
Kidney loop of Henle epithelial cell
Trophoblast cell
Erythroid lineage cell
Myometrial cell
Melanocyte
Club cell
Mast cell
Pericyte cell
Inhibitory neuron
Acinar cell
Excitatory neuron
Plasma cell
General_cell_type

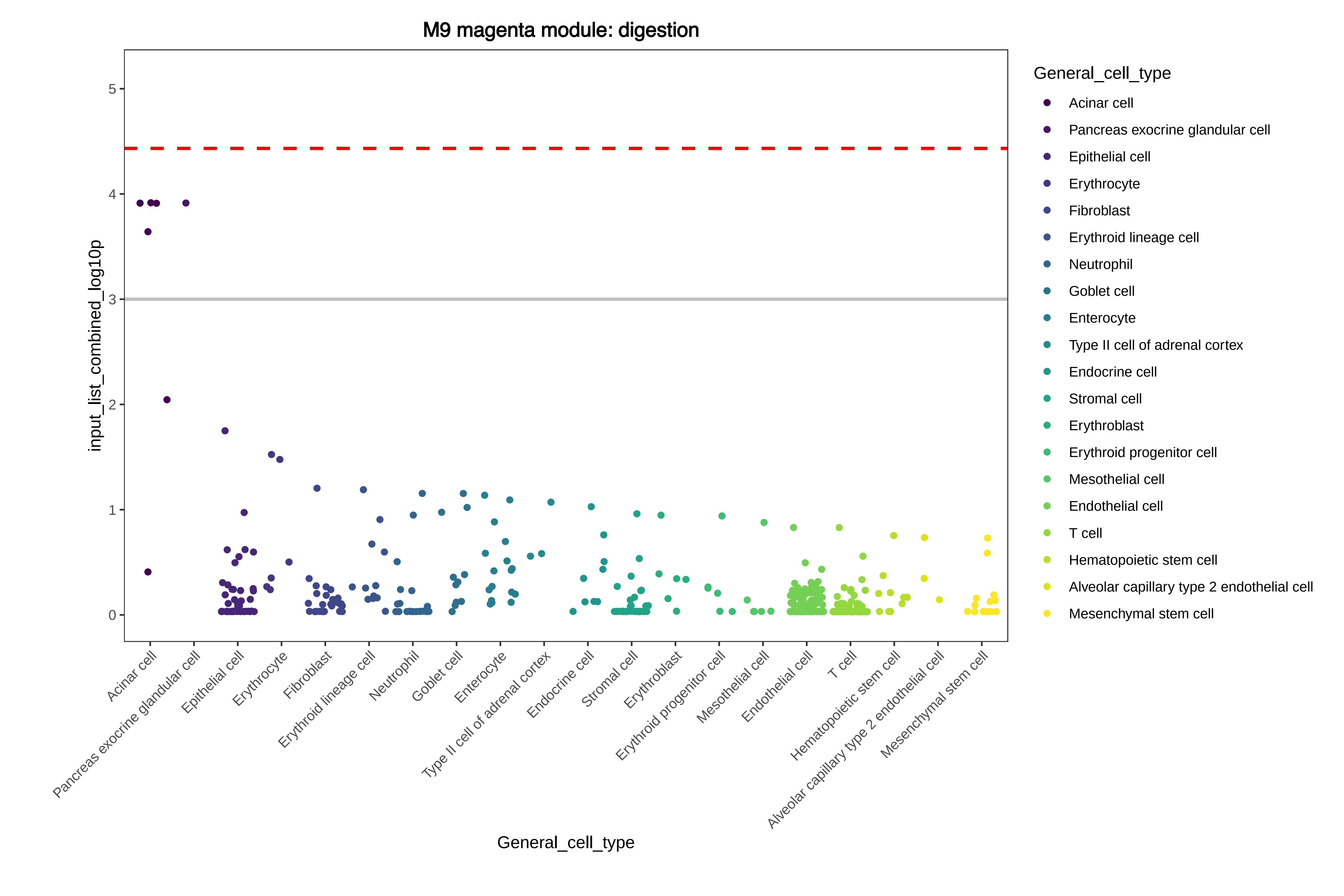
M9 magenta module: digestion
input_list_combined_log10p
0
1
2
3
4
5
General_cell_type
Acinar cell
Pancreas exocrine glandular cell
Epithelial cell
Erythrocyte
Fibroblast
Erythroid lineage cell
Neutrophil
Goblet cell
Enterocyte
Type II cell of adrenal cortex
Endocrine cell
Stromal cell
Erythroblast
Erythroid progenitor cell
Mesothelial cell
Endothelial cell
T cell
Hematopoietic stem cell
Alveolar capillary type 2 endothelial cell
Mesenchymal stem cell
General_cell_type
